# Awake Fibreoptic Intubation in the Sitting Position in a Patient with a Huge Goitre

**DOI:** 10.1155/2011/352672

**Published:** 2011-11-22

**Authors:** Kirti Nath Saxena, Sudhir Kumar, Bharti Taneja, Prachi Gaba

**Affiliations:** Department of Anesthesiology, Maulana Azad Medical College and Associated Hospitals, B-302 Geetanjali Apartments, Vikas Marg Extension, New Delhi 110092, India

## Abstract

A 46-year-old woman was anesthetized for total thyroidectomy. The thyroid was massive, deviating the trachea to the right and causing attenuation of the trachea radiologically. She had symptoms of respiratory obstruction in the supine position. Awake FOB-guided intubation was done in sitting position after airway topicalisation, and the airway was intubated with difficulty with 7.0 mm cuffed orotracheal tube. We describe this case in detail and discuss the significance of careful approach to planning and preparation in the management of such a case.

## 1. Case Report

**Figure 1:**
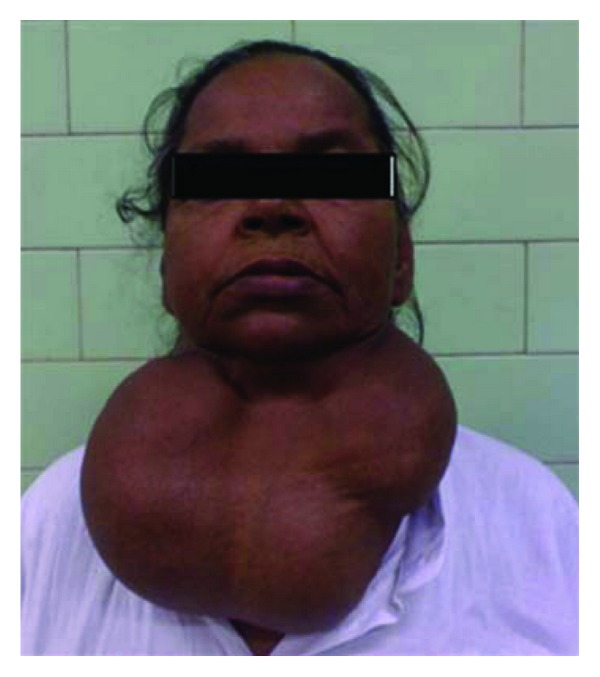
Patient with huge thyroid.

**Figure 2:**
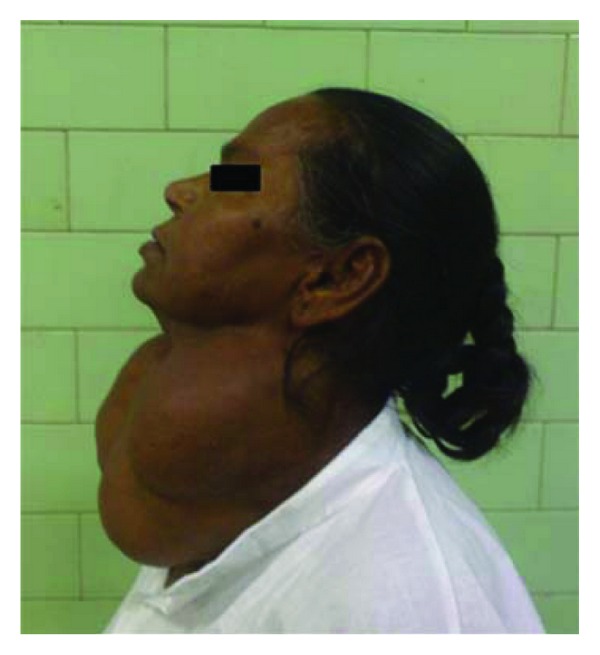
Lateral view of patient.

A 46-year-old female patient weighing 52 kg presented with a very large multinodular goitre for total thyroidectomy. She had no other systemic disease. The thyroid swelling was 30 × 30 × 20 cm in size with a lobulated surface extending from the lower jaw to below the sternal notch (see Figures 1 and 2). There were no distended veins on the chest or retrosternal extension, but the patient gave a history of difficulty in breathing on lying in the supine position. On examination it was also found that she became breathless on lying down and was unable to maintain the supine position. This was attributed to the pressure effect of the large thyroid. There were no other pressure symptoms or complaints, and the patient was clinically and biochemically euthyriod.

Indirect laryngoscopy revealed normal vocal cord mobility. Airway examination showed adequate mouth opening, protruding incisors, modified Mallampati's class (MPC) lV, mild limitation of neck extension, and severely restricted neck flexion. Radiological examination of the swelling revealed attenuation of airway with lateral displacement of the trachea to the right on anteroposterior view and compression of trachea with increased gap behind it on lateral view suggesting tracheomalacia.

Since the patient became breathless in the supine position, after considering various alternatives, an awake fibreoptic intubation in sitting position was planned for the patient. The procedure was explained to the patient, and she agreed to cooperate. On the day of surgery, no preoperative sedation was prescribed. Difficult airway management cart was kept ready, and the patient shifted on the OT table. Standard monitors (namely, ECG, Pulse Oximeter, NIBP) were attached and the baseline vitals recorded. Inj. glycopyrrolate 0.2 mg i.v. was administered. After nebulization with 5 mL of 4% lignocaine, the patient's airway was anesthetized with lignocaine viscous 2% gargles and lignocaine spray (10%) into the laryngopharynx. Fiberoptic bronchoscope (FOB) was loaded with a 7.0 mm armoured endotracheal tube and the patient made to sit on the operation table. The anesthetist stood on the left side of the patient facing the FOB monitor. After putting in a bite block, the bronchoscope was inserted orally and advanced towards laryngeal inlet. Laryngeal and esophageal openings were visualized side by side with the laryngeal inlet on the right side. Patient was instructed to take deep breaths to facilitate identification of the airway. FOB could be negotiated through the vocal cords with difficulty because of airway distortion after spraying lignocaine by “spray as you go” technique. The posterior wall of trachea was found to be bulging into the lumen of upper trachea. The fiberscope was advanced beyond this narrowing of the trachea and positioned above the carina. Endotracheal tube (ET) was then threaded over the FOB beyond the tracheal narrowing, under vision, and the FOB removed. The breathing circuit was attached, and the tube placement was confirmed by movement of reservoir bag and capnography. The patient was made to lie in the supine position and anesthesia induced with inj. thiopentone sodium 250 mg, Fentanyl 100 mcg, and, after confirming chest expansion, muscle relaxation was achieved with vecuronium bromide 5 mg. The ET was firmly secured and anesthesia maintained with O_2_ in N_2_O and isoflurane.

The intraoperative course was uneventful for the patient. Large venous channels were found in the gland intraoperatively, and the total blood loss was about 1000 mL, which was adequately replaced with crystalloids and colloids. The patient remained hemodynamically stable throughout the procedure.

Postoperatively, the patient was reversed with inj. Atropine 1.0 mg and inj. Neostigmine 2.5 mg i.v. after the return of spontaneous breathing efforts. FOB was passed through the swivel connector attached to ET and positioned just above the tip of the tube, which was then gradually withdrawn under vision. Mild tracheal lumen collapse was observed. The left vocal cord movement was found to be normal, but the right vocal cord was paralysed. Surgeons were informed about the same, and the patient was extubated when completely awake and responding to verbal commands. The postoperative course remained uneventful, and the patient was discharged after few days.

## 2. Discussion

Difficulty with intubation may be caused by an enlarged thyroid gland producing tracheal deviation, compression, or both [1, 2]. Tracheal intubation in a patient with tracheal deviation or compression is challenging for the anesthetist. The distorted airway makes orotracheal intubation with traditional laryngoscopy difficult. In a study to predict difficult tracheal intubation in thyroid surgeries, Bouaggad et al. [3] found that there was significantly increased incidence of difficulty in endotracheal intubation with tracheal deviation, tracheal compression, presence of dyspnea, Mallampati class III/IV airway and with neck mobility <90°. Moreover, induction of general anesthesia could be risky because it may precipitate complete airway closure and make facemask ventilation and tracheal intubation impossible. Another concern is tracheomalacia in these patients, which can complicate both intubation and extubation. Pressure on trachea exerted by a long-standing neck mass could have caused necrosis to the parts of tracheal wall which can lead to complete collapse of the airway with muscle relaxation. Fibreoptic intubation which can safely and promptly secure the airway has been recommended for the airway management in patients with difficult airways and should be considered as an early option in airway management in patients with tracheal deviation or compression from large thyroid mass [4]. Fiberoptic intubation has been reported successfully in patients with enlarged thyroids [5, 6] in a difficult airway situation. In our case, apart from the history of respiratory difficulty on lying down, there was radiological evidence of tracheal deviation and compression. In addition to these, the patient had prominent incisors, restricted neck flexion, and extension and was Mallampati's class IV airway. In order to avoid airway problems during endotracheal intubation, the anesthetist should carefully perform preoperative evaluation and have a plan for airway management in advance in the lying down as well as sitting position [7]. In our case, since there were multiple factors that could have precipitated problems in airway management, several options were weighed, and awake fibreoptic guided tracheal intubation in sitting position was planned. Sitting position also has the advantage of facilitating visualization by gravity drainage of secretions and movement of the tip of the fiberscope downwards towards the larynx without much manipulation. Fibreoptic intubation was considered as an early option because the chances of successful intubation are higher before excessive bleeding and edema have occurred [8]. Also we gave no sedation to the patient as she gave history suggestive of airway obstruction. Complete upper airway obstruction as a result of laryngospasm due to inadequate topicalization of the airway and additional sedation given in the operating room has been reported [9].

 This paper describes the successful use of early FOB guided awake intubation in sitting position in a patient with a massive goitre. Fibreoptic bronchoscope was also helpful in visualization of the airway during extubation.
